# Protein acetylation in mitochondria plays critical functions in the pathogenesis of fatty liver disease

**DOI:** 10.1186/s12864-020-06837-y

**Published:** 2020-06-26

**Authors:** Zhang Le-tian, Hu Cheng-zhang, Zhang Xuan, Qin Zhang, Yan Zhen-gui, Wei Qing-qing, Wang Sheng-xuan, Xu Zhong-jin, Li Ran-ran, Liu Ting-jun, Su Zhong-qu, Wang Zhong-hua, Shi Ke-rong

**Affiliations:** Shandong Key Laboratory of Animal Bioengineering and Disease Prevention, College of Animal Science and Technology, Shandong Agricultural University, No. 61 Daizong Street, Taian, Shandong 271018 P. R. China

**Keywords:** Acetylome, Lipid metabolism, Fatty liver, Dairy cattle, Perinatal period

## Abstract

**Background:**

Fatty liver is a high incidence of perinatal disease in dairy cows caused by negative energy balance, which seriously threatens the postpartum health and milk production. It has been reported that lysine acetylation plays an important role in substance and energy metabolism. Predictably, most metabolic processes in the liver, as a vital metabolic organ, are subjected to acetylation. Comparative acetylome study were used to quantify the hepatic tissues from the severe fatty liver group and normal group. Combined with bioinformatics analysis, this study provides new insights for the role of acetylation modification in fatty liver disease of dairy cows.

**Results:**

We identified 1841 differential acetylation sites on 665 proteins. Among of them, 1072 sites on 393 proteins were quantified. Functional enrichment analysis shows that higher acetylated proteins are significantly enriched in energy metabolic pathways, while lower acetylated proteins are significantly enriched in pathways related to immune response, such as drug metabolism and cancer. Among significantly acetylated proteins, many mitochondrial proteins were identified to be interacting with multiple proteins and involving in lipid metabolism. Furthermore, this study identified potential important proteins, such as HADHA, ACAT1, and EHHADH, which may be important regulatory factors through modification of acetylation in the development of fatty liver disease in dairy cows and possible therapeutic targets for NAFLD in human beings.

**Conclusion:**

This study provided a comprehensive acetylome profile of fatty liver of dairy cows, and revealed important biological pathways associated with protein acetylation occurred in mitochondria, which were involved in the regulation of the pathogenesis of fatty liver disease. Furthermore, potential important proteins, such as HADHA, ACAT1, EHHADH, were predicted to be essential regulators during the pathogenesis of fatty liver disease. The work would contribute to the understanding the pathogenesis of NAFLD, and inspire in the development of new therapeutic strategies for NAFLD.

## Background

More than 60% of dairy cows develop fatty liver during the transition period from dry milk to lactation due to negative energy imbalance [1], resulting in weakened liver function and decreased milk production [2]. The fatty liver disease in dairy cows is a typical type of nonalcoholic fatty liver disease (NAFLD), mainly caused by obesity and stress response. In the first month after delivery, 5–10% of dairy cows had a severe form of fatty liver, and 30–40% had mild or moderate fatty liver [3]. The occurrence of fatty liver in dairy cows leads to huge economic losses, not only because of decreased milk production but also because of prolonged calving intervals and weakened reproductive performance [4, 5], and therefore shortened their service life.

As an important type of protein post-translational modification, lysine (K) acetylation modification can change the protein-protein interactions, protein homeostasis, catalytic activity, and subcellular localization of metabolic enzymes [6, 7], as well as affect the structure of cell chromatin or activate transcriptional regulators in the nucleus [8]. Acetylation plays particular important roles in material and energy metabolism by modifying the activity and/or specificity of certain enzymes and substrates, thereby regulating glucose [9–11], lipid, and amino acid metabolism. Human-related studies have indicated that the change in protein acetylation pattern is associated with the occurrence and/or development of metabolic-related diseases such as obesity, cardiovascular disease, diabetes, and tumorigenesis [12, 13]. In particular, in livers, it has been predicted that acetylation modification is involved in most metabolic pathways by regulating glycolipid metabolism and urea cycles [14]. Fatty liver disease in dairy cows is a type of metabolic disorder. Little is known about the pathogenesis of perinatal fatty liver in dairy cows [15]. The objective of the present study is to investigate the possible role of protein acetylation in liver function during the transition period from dry milk to lactation in dairy cattle.

TMT labeling technology is a peptide in vitro labeling technology developed by Thermo Scientific, USA. The technology uses ten isotopic labels to label the amino groups of the peptide. After LC-MS/MS analysis, the relative content of protein in ten different samples can be compared simultaneously. TMT technology is a commonly used differential proteomics technology, which is widely used in the field of disease marker screening, drug targets, animal disease resistance/anti-stress mechanisms, animal and plant development, and differentiation mechanisms. The TMT labeling technology has the advantages of high sensitivity, wide application range, fast analysis speed, and good separation effects.

In this study, we focused on the protein acetylation modification in liver tissue of cows with severe fat deposition, and healthy livers. In this project, differentially acetylated proteins were identified through bioinformatics analyses which were carried out by combing a series of advanced technologies, such as TMT-labeled acetylated peptide enrichment and mass spectrometry-based quantitative proteomics. This study reveals a comprehensive acetylome profiling of fatty liver disease in dairy cattle and identifies potential biomarkers based on protein acetylation level. These results provide a strong foundation for further understanding of important protein regulatory targets in the development of NALFD in human beings and/or animals.

## Results

### Overview of acetylation

**Fig. 1:**
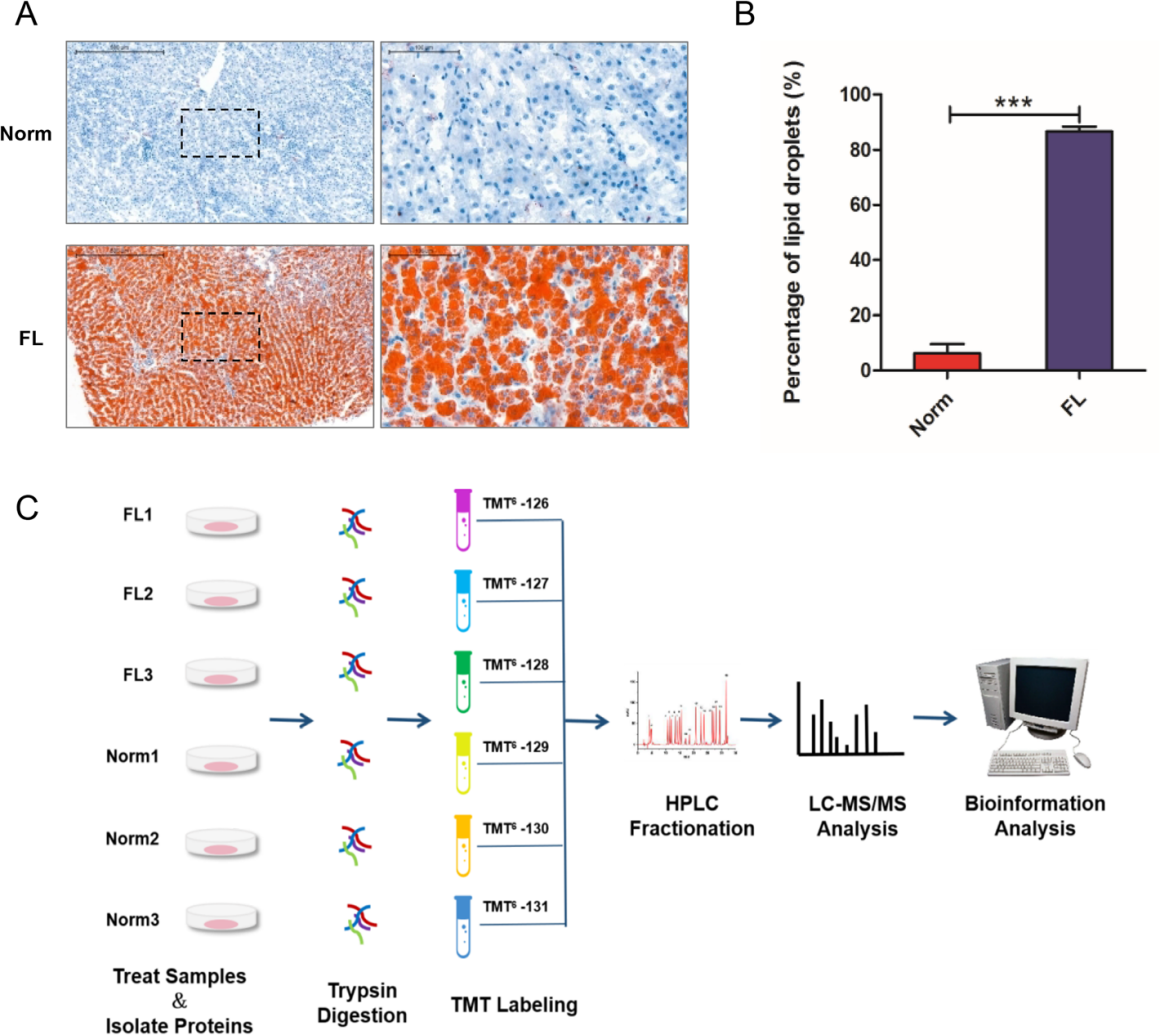
Liver sample selection and study design. **a** Representative hepatic histology sections of liver tissues from dairy cows during partuition period. Oil Red O staining assay was used for fat content assessment in hepatic cells, therefore classified into normal liver (Norm) and fatty liver (FL), scale bar 500 μm. Blue dots indicate cell nucleus. Brown or red indicate lipid drops in cells that disovled Oil red. The right panel is a high-powered magnification of the black dashed area in the left panel. **b** Comparison of average percentage of hepatocytes containing lipid droplets in liver from Norm (*n* = 6) and FL (*n* = 8) groups. ***P < 0.001. **c** The whole experimental work-flow for the study

The tissues of normal (Norm1, Norm2, Norm3) and fat-deposited (FL1, FL2, FL3) livers were obtained for acetylome profiling. Oil red O staining results of the liver tissue samples showed that there was a significant difference between the fatty liver group (86.75% ± 4.83%, *n* = 6) and the normal liver group (6.26% ± 5.23%, *n* = 8) (Fig. 1a, b). The experimental workflow of this study is shown in Fig. 1c. The thermal maps of Pearson correlation coefficients, calculated upon log 2 logarithm conversion of the relative peptide quantitative values so as to obey the normal distribution, between two group samples were averaged at 0.74 in the Norm group and 0.80 in the FL group, indicating that the biological replicates within the group met the standard of quantitative consistency (Fig. S1A). Additionally, the distribution of peptide mass errors is close to zero, and most of them are less than 5 ppm (Fig. S1B). Moreover, the length of the peptide segments showed a theoretical distribution (Fig. S1C). These results indicated appropriate sample preparation and credible data quality.

**Fig. 2:**
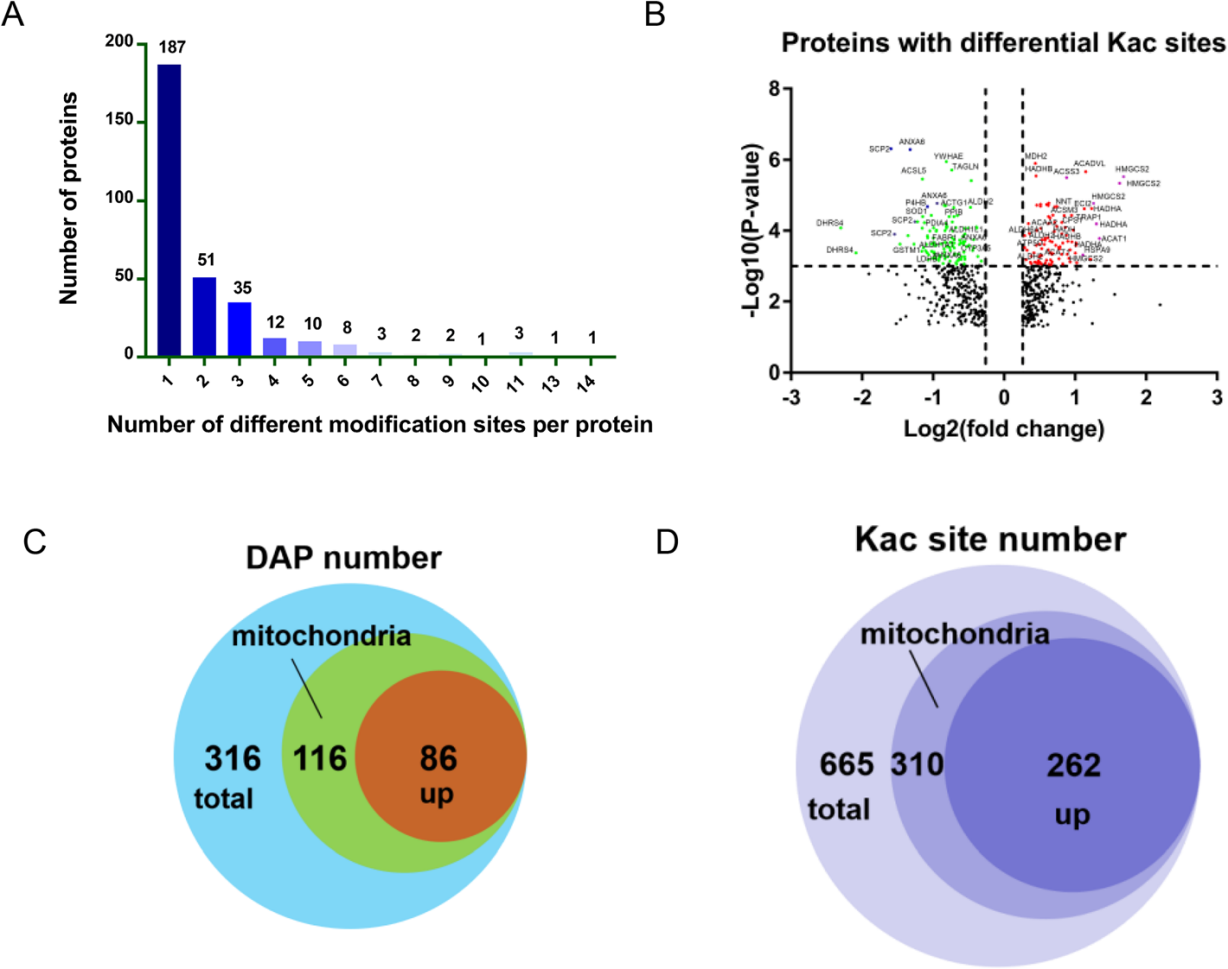
The identified differentially lysine acetylated (Kac) proteins and/or sites mainly localized to mitochondria. **a** Number distribution of different Kac sites on proteins. **b** Volcano plot of statistical significance against log2-fold change between the Norm group (*N* = 3) and FL group (*N* = 3), showing significantly differentially expressed proteins colored in green and red. **c** Among the identified differentially acetylated proteins (DAPs), the DAPs with higher acetylation levels mainly located in mitochondria, account for a large proportion (86/116) of all identified DAPs. **d** Among the identified differentially Kac sites, the up-regulated Kac sites located in the mitochondria, account for a large proportion (310/335) of all identified Kac sites

In total, 1841 differential acetylation sites on 665 proteins were identified. Among them, 1072 sites on 393 proteins were quantified, 307 sites on 122 proteins were significantly higher acetylated (fold-change> 1.2, *P* < 0.05), and 358 sites on 213 proteins were significantly lower acetylated (fold-change< 1/1.2, *P* < 0.05). Among them, 19 proteins are both higher acetylated and/or lower acetylated at different positions (Table 1, Table S1). The number of acetylation sites on the acetylated proteins was different (Fig. 2a). The number of proteins with only one acetylation site was more than half (59.2%, 187/316). The proportion of proteins with two, three, and more than four acetylation sites was 16.1% (51/316), 11.1% (35/316), and 13.6% (43/316), respectively. There were 21 proteins containing > 6 Kac sites of which HADHA, ANXA6, CPS1, GOT2, HMGCS2, and ACAT1 are highly acetylated (Table 2). Significance analysis showed that differentially acetylated proteins (DAPs) enriched in fatty acid oxidation pathway were significantly higher acetylated, such as HADHA, HADHB, ACAA2, ACADM, and ACADVL. DAPs promoting ketosome synthesis and enzymes involved in energy metabolism were also significantly higher acetylated, such as HMGCS2, ACAT1, PCK2, IDH2, MDH2, and SUCLG1. Proteins that are molecule transport-related were significantly lower acetylated, such as FABP1, ANXA6, and SCP2 (Fig. 2b). These results suggest that the metabolites transport in the liver tissue with fat deposition significantly inhibited. Although energy metabolism and fatty acid oxidation were enhanced, the accumulation of fat in the liver was unavoidable. In addition, among all the identified DAPs, it was found that 36.7% (116/316) were localized to mitochondria, with 74.1% (86/116) of these proteins higher acetylated (Fig. 2c). Moreover, as for all the Kac sites, 46.6% (310/335) of them were again localized in the mitochondria, with 84.5% (262/310) of these sites higher acetylated (Fig. 2d). This suggests that proteins that were associated with mitochondrial function were critical for the liver metabolism, and protein acetylation played an essential role during the development of fatty liver disease in dairy cattle.

**Table 1:** Statistical results of differentially lysine acetylated (Kac) sites and proteins

Items	Identified	Quantified	Higher acetylated (> 1.2)	Lower acetylated (< 1/1.2)
**Kac sites**	1841	1072	307	358
**Proteins**	605	393	122	213

**Table 2:** The background information and their distribution of differentially acetylated sites at lysines in proteins

Protein accession number	Protein name	Number of potential modification sites	Number of differentially acetylated sites	Position	Modified sequence^**a**^	Average fold change of acetylated level by FL/Norm	Maximum ***P*** value^b^
Q3SZ00	HADHA	23	14	516	MQLLEIITTEK(1)TSK	1.50 ± 0.49 (*n* = 14)	≤0.0469
P79134	ANXA6	17	13	306	SLYSMIK(1)NDTSGEYK	0.59 ± 0.11 (*n* = 13)	≤0.0473
F1ML89	CPS1	35	11	875	LTSIDK(1)WFLYK	1.31 ± 0.25 (*n* = 11)	≤0.0179
P12344	GOT2	16	11	90	K(1)AEAQIAAK(1)NLDK	1.31 ± 0.25 (*n* = 11)	≤0.0354
Q2KIE6	HMGCS2	13	11	350	LEDTYTNK(1)DVDK(1)AFLK	1.96 ± 0.82 (*n* = 11)	≤0.0496
Q29RZ0	ACAT1	14	10	197	IHMGNCAENTAK(1)K	1.86 ± 0.38 (*n* = 10)	≤0.0169
A0A140T871	GLUD1	18	9	460	LTFK(1)YER	1.42 ± 0.30 (*n* = 9)	≤0.0495
F1N7K8	ALDH6A1	10	9	333	K(1)WLPELVER	1.34 ± 0.30 (*n* = 9)	≤0.0246
F1MQV8	ACSS3	13	8	124	HIENGK(1)GDK	1.68 ± 0.24 (*n* = 8)	≤0.0083
Q3ZCH0	HSPA9	10	8	612	LK(1)EEISK	1.41 ± 0.43 (*n* = 8)	≤0.0302
Q3T0R7	ACAA2	11	7	240	QTMQVDEHPRPQTTMEQLNK(1)LPPVFK(1)K	1.47 ± 0.41 (*n* = 7)	≤0.0416
F1MV74	SCP2	9	7	189	NHK(1)HSVNNPYSQFQK	0.37 ± 0.07 (*n* = 7)	≤0.0017
Q5E9F8	H3F3A	8	7	28	K(1)SAPSTGGVK(0.857)K(0.143)PHR	0.58 ± 0.06 (*n* = 7)	≤0.0426
Q32LG3	MDH2	14	6	328	ASIK(1)K(1)GEEFVK	1.53 ± 0.21 (*n* = 6)	≤0.0017
F1N206	DLD	12	6	159	ITGK(1)NQVTATK	1.26 ± 0.36 (*n* = 6)	≤0.0191
P05307	P4HB	11	6	387	NFEEVAFDEK(1)K	0.56 ± 0.06 (*n* = 6)	≤0.0151
P20000	ALDH2	11	6	371	TEQGPQVDETQFK(1)K	1.26 ± 0.29 (*n* = 6))	≤0.0176
O46629	HADHB	10	6	189	MMLDLNK(1)AK(1)TLAQR	1.43 ± 0.34 (*n* = 6)	≤0.0350
Q0VCM4	PYGL	9	6	465	IHSDIVK(1)TQVFK	0.61 ± 0.08 (*n* = 6)	≤0.0433
F1MZP8	_	7	6	348	LVTDFMAK(1)K	0.70 ± 0.06 (*n* = 6)	≤0.0358
P52898	_	7	6	161	DAGLTK(1)SIGVSNFNHK	0.63 ± 0.09 (*n* = 6)	≤0.0217

^a^ Shown is the distribution range of *P*-values of all the aceltylated sites in this protein by picking up the ultimate value

^b^ Identified peptide sequence containing acetylated modification sites marked with localization and probabilities

### Analysis of Kac motifs

**Fig. 3:**
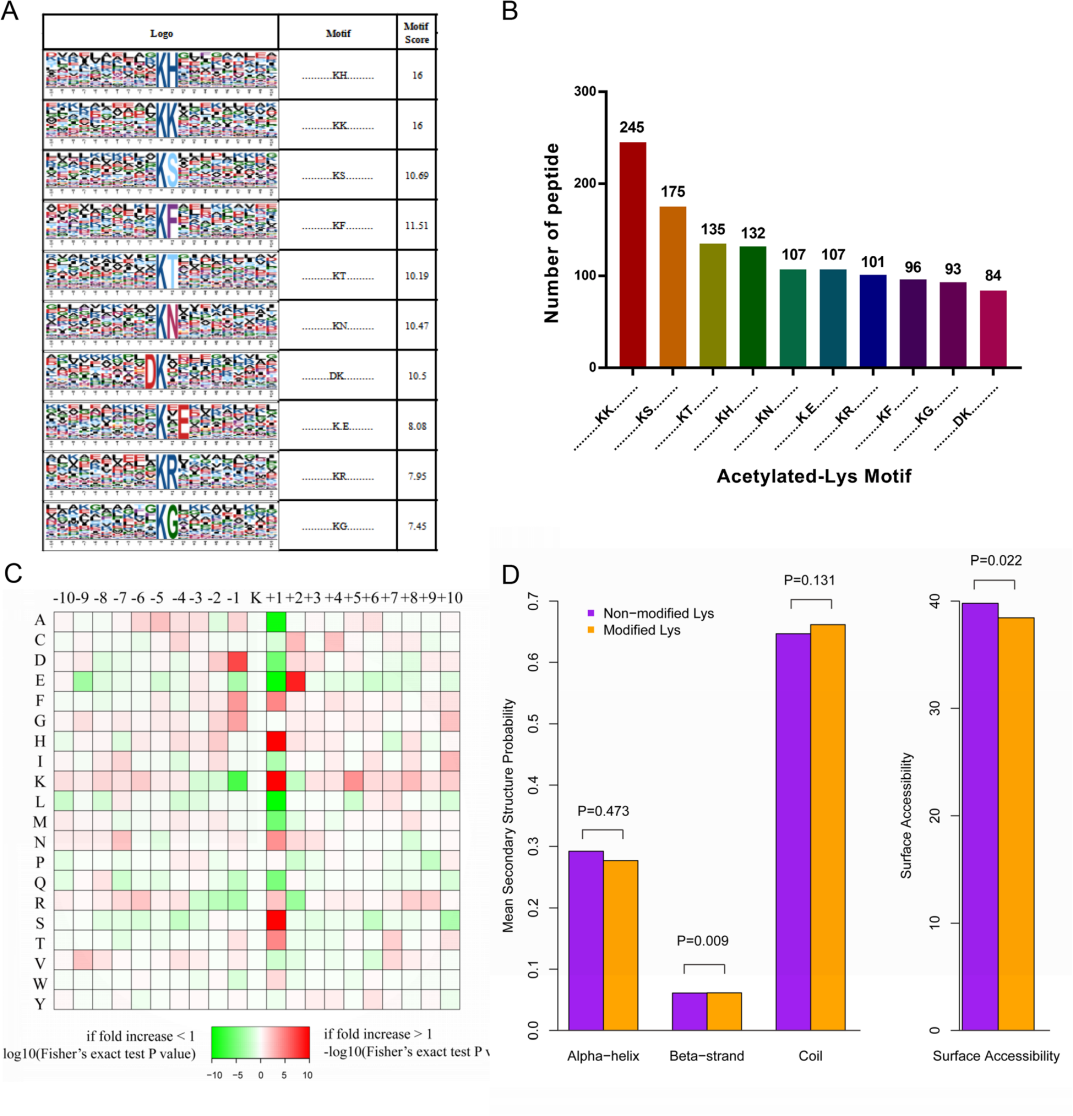
Motif analysis of the identified acetylation peptides. **a** Probable sequence motifs of acetylation sites in fatty liver tissues identified using Motif-X. **b** Number of identified peptide containing acetylated lysines and their probable motifs. **c** Heat map showing the relative frequencies of amino acids in specific positions, including enrichment (red) or depletion (green) of amino acids flanking the acetylated lysine in fatty liver proteins. **d** Location probabilities of acetylated and/or non-acetylated lysines in protein secondary structures (alpha-helix, beta-strand, and coil) and surface accessibility

To explore the preference for lysine acetylation sites, motif-x was used to detect the amino acid occupancy frequency at the location around the identified modification sites. These motifs exhibit different abundances (Fig. 3a), with the KacK (19.2%, 245/1275), KacS (13.7%, 175/1275), KacT (10.6%, 135/1275), and KacH (10.4%, 132/1275) motifs being the most common (Fig. 3b, c). The results of the motif analysis showed that the residues of histidine (H), lysine (K), and serine (S) were highly enriched at the + 1 position near the Kac site, and the aspartic acid (D) was observed at the − 1 position. Glutamate (E) enrichment was observed at the + 2 position (Fig. 3c).

Structural analysis of proteins containing lysine was performed using NETSURFP software, so as to understand the locations of acetylated and/or non-acetylated lysine in the secondary structures of proteins (alpha-helix, beta-strand and coil). Results indicated that significantly less acetylated sites were in the beta-strand (*P* = 0.009) or surface-accessible (*P* = 0.022) than non-acetylated sites (Fig. 3d). However, for lysine located in the alpha-helix and/or coil region, there was no statistical difference between acetylated and/or non-acetylated lysine.

### Functional enrichment analysis of differentially acetylated proteins

**Fig. 4:**
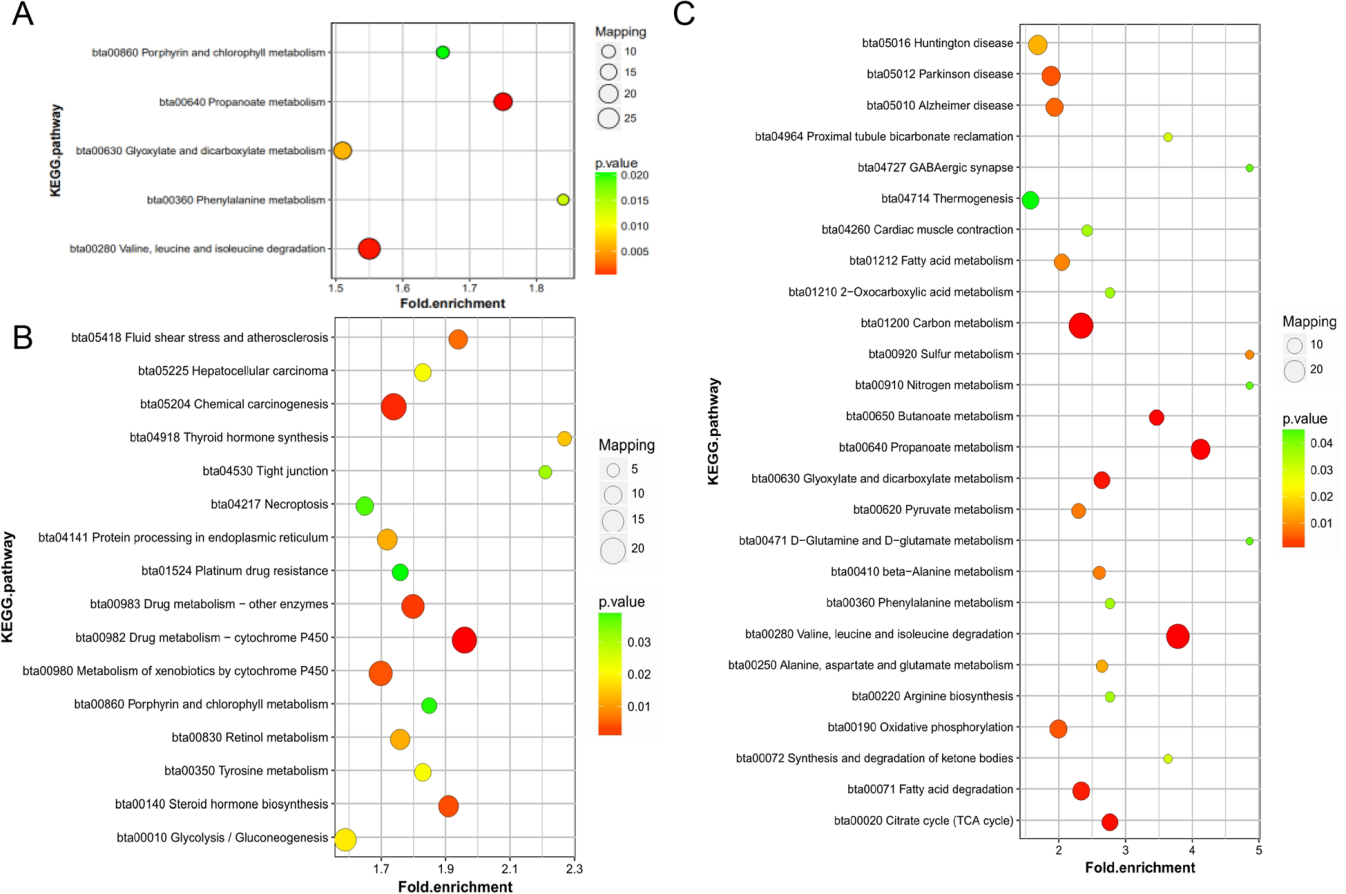
KEGG pathway annotation of differentially expressed Kac proteins. **a** The identified Kac proteins was significantly enriched in metabolism-related pathways. **b** KEGG pathways enriched by lower-acetylated proteins, they were involved in substance metabolism, such as glycolysis/gluconeogenesis in hepatocytes. **c** KEGG pathway enriched by higher-acetylated proteins, they were energy/amino acid metabolism-related and/or biosynthesis pathways-related

To further understand the functions and features of the identified differentially modified proteins, functional enrichment and cluster analysis were performed. Gene Ontology (GO) analysis was carried out and assessed the biological processes, molecular functions, and cellular components of these identified proteins to attend. The Kac proteins were all cellular component located in cell, organelle, membrane and/or extracellular region (Fig. S2A). Mitochondria and cytoplasm are the main distributed areas in the cell for the Kac proteins (Fig. S2B). The identified Kac proteins mainly belonged to the metabolic, cellular, and single biological processes and biological regulation (Fig. S2C). Binding and catalytic activity are the major functions of the identified Kac proteins (Fig. S2D). Actually, the KEGG pathway enrichment analysis showed that Kac protein was mainly involved in propanoate metabolism, valine, leucine, and isoleucine degradation, and glyoxylate and dicarboxylate metabolism (Fig. 4a). Lower-acetylated proteins are significantly enriched in pathways such as substance metabolism, protein processing and/or glycolysis/gluconeogenesis in hepatocytes (Fig. 4b), while higher-acetylated proteins are significantly enriched in energy/amino acid metabolism-related and/or biosynthesis pathways (Fig. 4c). These results suggest that acetylation modification mainly modulates the cellular biological processes that are closely associated with mitochondria function, which is critical to the energy metabolism in liver. In another word, the pathogenesis of fatty liver disease in dairy cows were presumed to be closely related to the dysfunction of mitochondrial metabolism, and lysine acetylation of target proteins could be one of the pivotal modification manners during the process.

**Fig. 5:**
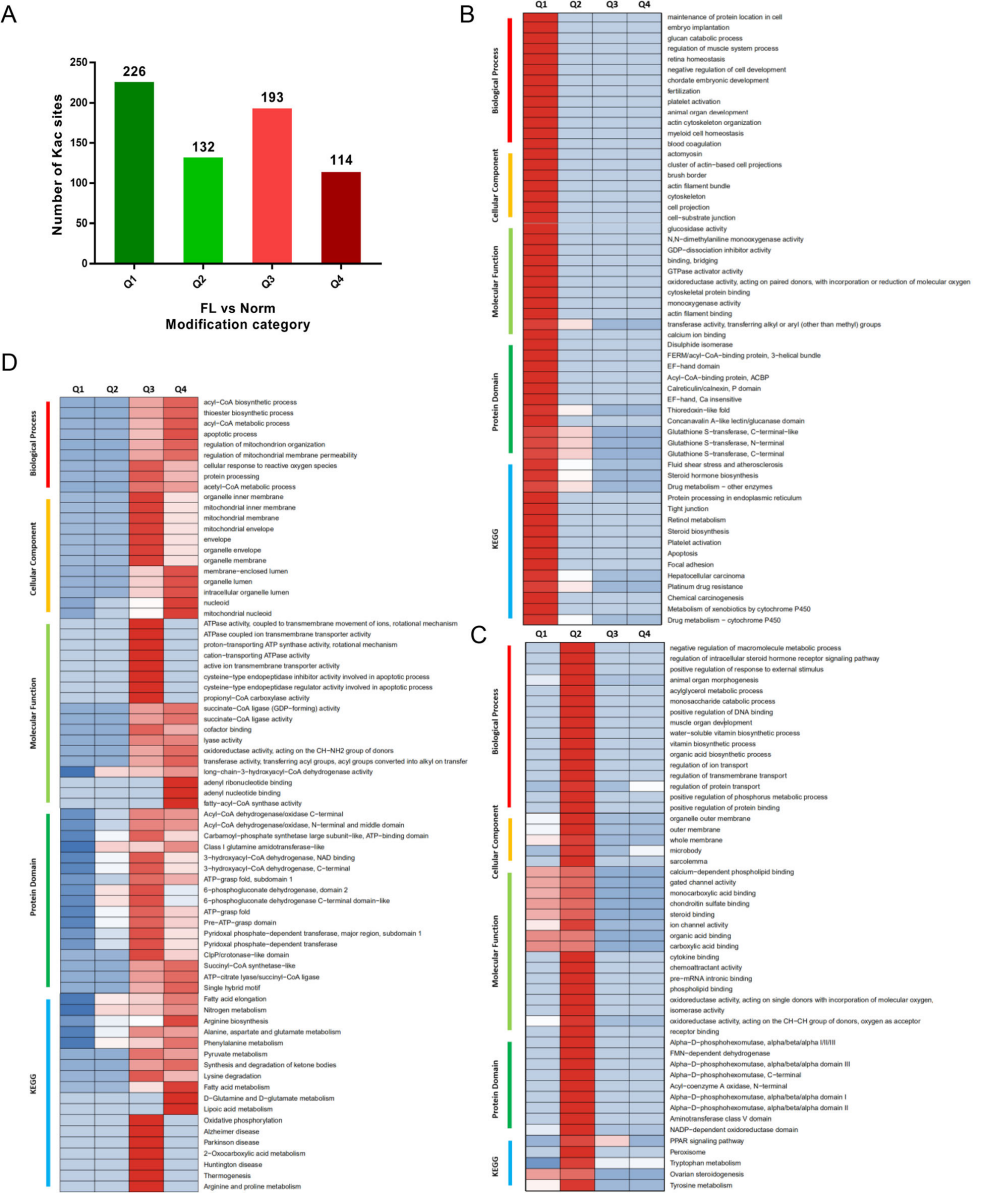
Cluster analysis of differential Kac proteins based on their biological processes, cellular components, molecular functions, protein domains and/or enriched KEGG pathways. **a** Distribution of differential acetylation sites as per their fold changes. The differentially expressed proteins were classified into four groups according to their differentially expressed folds: Q1 (0 < Ratio ≤ 1/1.5), Q2 (1/1.5 < Ratio ≤ 1/1.2), Q3 (1.2 < Ratio ≤ 1.5) and Q4 (Ratio > 1.5). **b** Cluster analysis of the DAPs classified into the Q1 class. **c** Cluster analysis of DAPs classified into Q2 class. **d** Cluster analysis of DAPs classified into Q3 and Q4 class

According to the modification levels of the acetylated proteins, they were classified into four parts according to their fold changes (Fig. 5a): Q1 (226 DAPs, 0 < Ratio ≤ 1/1.5), Q2 (132 DAPs, 1/1.5 < Ratio ≤ 1/1.2), Q3 (193 DAPs, 1.2 < Ratio ≤ 1.5) and Q4 (114 DAPs, Ratio > 1.5). Then, enrichment analysis of GO, KEGG, and protein domains for proteins in each Q group were performed, Results indicated that the higher and lower Kac proteins enriched in distinct biological processes, cell components, molecular functions, protein domains, and/or KEGG pathways. Acetylated proteins in Q1 class (Fig. 5b), which are extremely downregulated, are widely involved in multiple biological processes and pathways, mainly including drug metabolism - cytochrome P450, metabolism of xenobiotics by cytochrome P450, apoptosis, chemical carcinogenesis, hepatocellular carcinoma, and steroid biosynthesis. Lower-acetylated proteins in Q2 class mainly localize in membranes (Fig. 5c) and participate in the regulation of macromolecule metabolism and protein, transmembrane, and ion transport that are related to ion upchannels and gated channels. These acetylated proteins are significantly involved in the PPAR signaling pathway and the amino acid metabolism pathway, such as the tyrosine and tryptophan metabolism pathway. Therefore, it suggests that the downregulation of protein acetylation was mainly involved in protein transport, cell communication and steroid biosynthesis, causing dysfunction in cell apoptosis and cell transport, thereby pathological metabolism in hepatocytes.

The proteins with higher acetylation levels in Q3 and Q4 class got overlapping enrichment preference (Fig. 5d). Both Q3 and Q4 proteins are mitochondria-associated and participate in multiple acyl-CoA metabolic processes by altering the activities of acyl-CoA synthetase, acyl-CoA dehydrogenase and acyl-CoA transferase. The KEGG pathway analysis showed that these acetylated proteins were enriched in fatty acid metabolism, pyruvate metabolism, ketone synthesis and degradation, and multiple amino acid metabolism pathways, such as D-glutamine and D-glutamate metabolism, lysine degradation, phenylalanine metabolism, alanine, aspartate and glutamate metabolism, and arginine biosynthesis (Fig. 5d). These data suggest that the upregulation of protein acetylation might significantly affect energy metabolism pathways, especially tricarboxylic acid cycle that are involved the hepatic mitochondrial function, and thereby causing amino acid and lipid metabolic disorders.

**Fig. 6:**
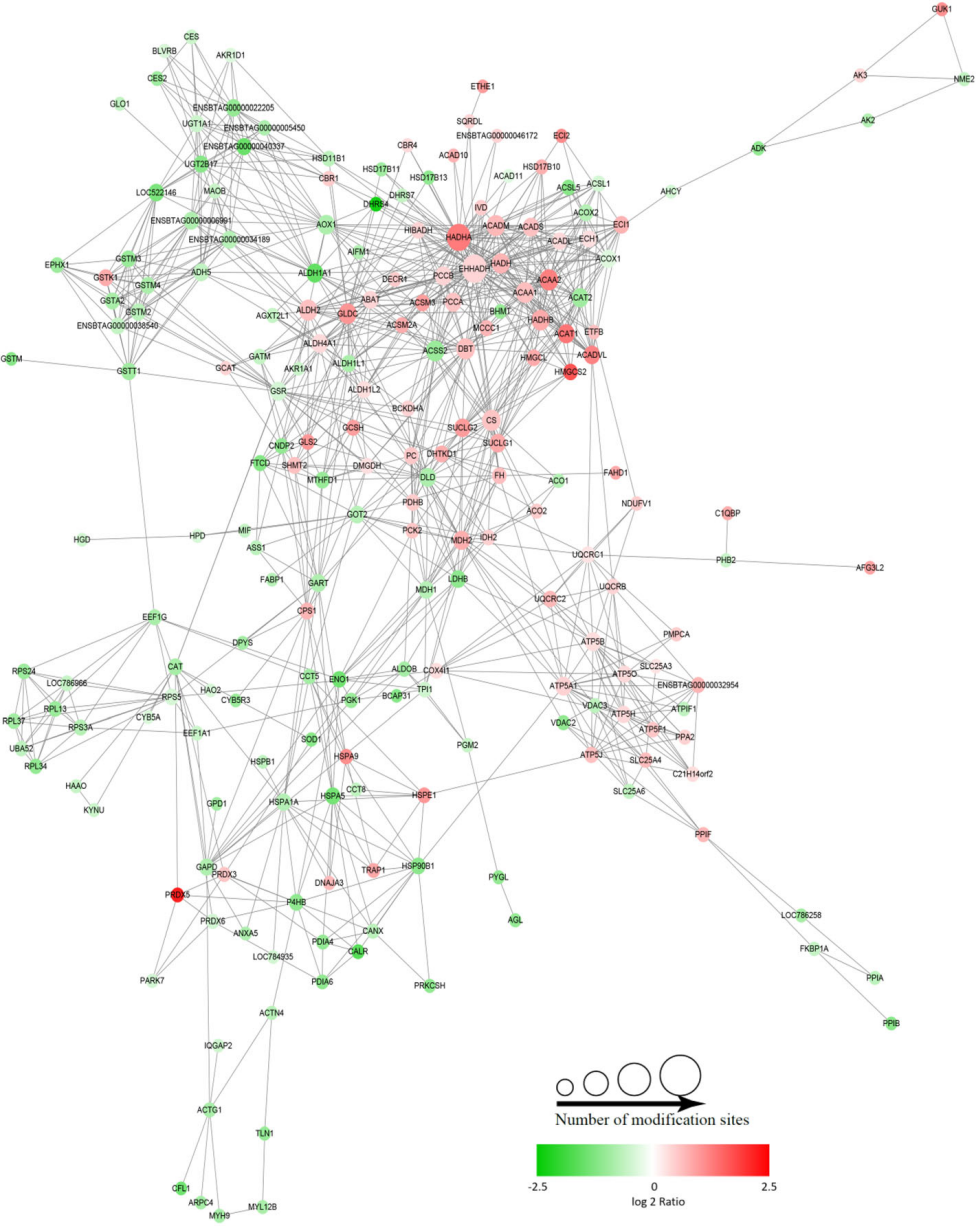
Identification of highly enriched protein-protein interaction clusters. In the interaction network, each acetylated protein was represented by a circle. The size of the circle indicates the number of acetylation sites in the protein. The color of the circle indicates the modified directions of the acetylated sites in the protein, red presents higher-acetylated sites and greed presents lower-acetylated sites

Additionally, these acetylated proteins are predicted to form protein-protein interaction network, with 209 nodes and 975 interactions (Fig. 6) Notably, 15 proteins were found to be simultaneously higher acetylated and/or lower acetylated on a single target protein at different positions. Among interaction network, four highly interconnected protein clusters were highlighted via the MCODE algorithm, they are involved in valine, leucine, and isoleucine degradation, oxidative phosphorylation, chemical carcinogenesis and ribosomes (Fig. S3).

### Identification of potential important hepatic proteins as acetylation targets

Some of the identified differentially acetylated proteins are abundantly and frequently interacting with other proteins (Fig. 6), indicating their potential important function in maintaining normal liver metabolism. However, part of the acetylated proteins contain multiple modification sites. As for the acetylated sites in a single protein, some are significantly higher acetylated and some are significantly lower acetylated (Table 3), such as enoyl-CoA hydratase and 3-hydroxyacyl CoA dehydrogenase (EHHADH), hydroxyacyl-CoA dehydrogenase trifunctional multienzyme complex subunit alpha (HADHA), acetyl-CoA acetyltransferase 1 (ACAT1), etc. These proteins are all mitochondria-localized proteins, and they can interact with many other proteins and participate in multiple lipid metabolism-related pathways and/or tricarboxylic acid cycle (Table 3).

**Table 3:** Potentially essential proteins as acetylation targets for maintaining normal hepatic metabolism

Protein accession number	Protein name	All/Up/Down^**a**^	Protein subcellular localization	No. of interactive nodes in PPI^**b**^	No. of associated pathways	Signaling pathways
E1BMH4	EHHADH	2/1/1	mitochondria	53	11	Fatty acid degradation; Valine, leucine and isoleucine degradation; Tryptophan metabolism; Peroxisome; beta-Alanine metabolism; Butanoate metabolism; PPAR signaling pathway; Propanoate metabolism; Fatty acid metabolism; Lysine degradation; Carbon metabolism;
Q3SZ00	HADHA	14/12/2	mitochondria	48	11	Fatty acid degradation; Valine, leucine and isoleucine degradation; Tryptophan metabolism; beta-Alanine metabolism; Butanoate metabolism; Propanoate metabolism; Fatty acid elongation; Fatty acid metabolism; Biosynthesis of unsaturated fatty acids; Carbon metabolism; Lysine degradation;
Q29RZ0	ACAT1	10/10/0	mitochondria	20	12	Synthesis and degradation of ketone bodies; Fatty acid degradation; Valine, leucine and isoleucine degradation; Tryptophan metabolism; Butanoate metabolism; Terpenoid backbone biosynthesis; Propanoate metabolism; Glyoxylate and dicarboxylate metabolism; Pyruvate metabolism; Fatty acid metabolism; Lysine degradation; Carbon metabolism;
Q32LG3	MDH2	6/6/0	mitochondria	20	5	Glyoxylate and dicarboxylate metabolism; Cysteine and methionine metabolism; Pyruvate metabolism; Citrate cycle (TCA cycle); Carbon metabolism;
P12344	GOT2	11/10/1	mitochondria	14	11	2-Oxocarboxylic acid metabolism; Alanine, aspartate and glutamate metabolism; Biosynthesis of amino acids; Arginine biosynthesis; Tyrosine metabolism; Arginine and proline metabolism; Phenylalanine, tyrosine and tryptophan biosynthesis; Fat digestion and absorption; Cysteine and methionine metabolism; Carbon metabolism; Phenylalanine metabolism;
F1ML89	CPS1	11/10/1	mitochondria	12	5	Nitrogen metabolism; Alanine, aspartate and glutamate metabolism; Biosynthesis of amino acids; Arginine biosynthesis; Carbon metabolism;
Q2KIE6	HMGCS2	11/10/1	mitochondria	11	4	Synthesis and degradation of ketone bodies; Valine, leucine and isoleucine degradation; Butanoate metabolism; Terpenoid backbone biosynthesis;

^a^ Number of all differentially acetylated sites (All) in the protein, higher acetylated sites (Up) and lower acetylated sites (Down) in the protein, separated by slash

^b^ Number of the nodes that were showing the reciprocally interactive proteins in the protein-protein interaction (PPI) network obtained by STRING analysis (shown as in Fig. 6 and/or Fig. S3)

EHHADH is a dehydrogenase involved in the fatty acid β oxidation and is one of the four essential enzymes in the peroxisome β oxidation pathway. *Ehhadh* was downstreamly targeted by the transcription factor PPARα, which mediating the fatty acid beta-oxidation, associated with lipid catabolism. Results in the study show that EHHADH have two acetylation sites, there were 53 other proteins in the EHHADH’s interaction network (Fig. 6, Table 3), which was the most frequent interactive protein in the network. EHHADH can participate in all different important hepatic metabolism processes, such as fatty acid degradation, valine, leucine, and isoleucine degradation; tryptophan metabolism; peroxisome; beta-alanine metabolism; butanoate metabolism; PPAR signaling pathway; propanoate metabolism; fatty acid metabolism; lysine degradation; and carbon metabolism. HADHA, an alpha subunit of a mitochondrial protein, catalyzes the last three steps of mitochondrial beta-oxidation of long-chain fatty acids. The interaction network show that HADHA interacts with 48 proteins (Fig. 6, Table 3), such as lipid metabolism-related protein HADHB, ACAT2, ACAT1, ACADVL, ACADM, and EHHADH. HADHA is also involved in hepatic metabolism associated pathways, such as fatty acid metabolism and biosynthesis, carbon metabolism, and amino acid degradation. Similar to EHHADH and HADHA, ACAT1, an enzyme catalyzing acetyl-CoA production, also participates in mitochondria-associated metabolic pathways. This suggests dysfunction of these important proteins via acetylation modification might destroy the metabolic processes, such as tricarboxylic acid cycle, thereby contributing to the development of fatty liver disease in dairy cows.

## Discussion

### Fatty liver in dairy cows

The liver is the central organ regulating the metabolic balance of carbohydrate, fat, and protein in mammals [16–18]. The energy metabolism balance is particularly important during the parturition period for dairy cattle, and the energy and substance metabolism is point-focused on the liver [19–21]. In the postperinatal period, the lactation of the cows slowly increases and therefore increases the body lactose consumption easily causing the cow is susceptible to experiencing an insufficient sugar supply, thus promoting fat mobilization in liver [22–26]. On the one hand, a large amount of fat mobilization promotes gluconeogenesis, increases the blood sugar concentration, and alleviates the negative energy balance. However, on the other hand, the enhanced fat mobilization promotes the dramatic increase of non-esterified fatty acids (NEFA) in the liver [21]. These NEFAs are partly oxidized in the liver and partly re-esterified to synthesize triglycerides (TG), which is difficult to be transported out of the liver by very-low-density lipoprotein (VLDL) [27]. Especially for dairy cattle, the lack of adequate lipid and liver esterase in the liver limits the hydrolysis of TAG, which leads to the excessive accumulation of TAG in the liver [23, 28–30], therefore resulting in susceptibility to fatty liver disease. While, nonalcoholic fatty liver disease (NAFLD) occurring in human beings, metabolic disorder syndromes and obesity are also usually the main causes, with increased plasma insulin and fatty acid concentration, elevated fasting aminotransferase (AST/ALT) and/or triglycerides level, and also abnormal lipid accumulation in the liver [31, 32]. In addition, another of the most important risk factors is histological evidence of hepatic inflammation [33, 34] caused by acute inflammation and subacute inflammation [35]. Thus, dairy cows with fatty liver disease is a typical NAFLD animal model, good for revealing the pathology and pathogenesis of NAFLD [15]. The objective of the study is to reveal the molecular mechanism of pathogenesis for NAFLD by taking fatty liver diseased dairy cattle as research model, identifying potential important biological pathways and protein targets, and thereby further discovering the therapeutic targets.

### Liver acetylome profiling identified important biological pathways regulating the pathogenesis of fatty liver in dairy cows

Acetylome profiling of liver revealed the modification of protein acetylation in fatty livers (FL) was significantly different from normal (Norm) livers. Enrichment analysis of identified DAPs indicated that the protein modifications of the acetylation levels remarkably participated in the regulation of energy metabolic pathways, such as amino acid metabolism, carbon metabolism, citrate cycle, butanoate metabolism, glyoxylate and dicarboxylate metabolism, fatty acid degradation, pyruvate metabolism, and synthesis and degradation of ketone bodies (Fig. 4). Actually, the fatty liver disease occurred in dairy cows during their perinatal period is usually complicated by a high incidence of ketosis [36], with increased ketone body produced in the liver. All the possible dysregulated energy metabolic processes might be the direct cause for metabolism disorder and fat accumulation in livers of dairy cows. Moreover, the proteins in pathways that are closely associated with tricarboxylic acid cycle, such as valine, leucine, and isoleucine degradation, D-Glutamine and D-glutamate metabolism, lysine degradation, phenylalanine metabolism, alanine, aspartate, and glutamate metabolism, and arginine biosynthesis, also showed significantly increased acetylation levels (Fig. 5). It may be that the enhancements of the tricarboxylic acid cycle leads to an increase of α-ketoglutarate and oxaloacetic acid metabolism, which indirectly leads to the upregulation of these amino acid metabolism pathways. Higher and/or lower acetylated proteins are significantly enriched in pathways related to energy and substance metabolism and/or biosynthesis, drug metabolism and cancer related pathways (Fig. 4). Human nonalcoholic fatty liver disease (NAFLD) has four stages: simple steatosis, non-alcoholic steatohepatitis (NASH), cirrhosis, and hepatocellular carcinoma. Simple fatty liver is a benign stage of NAFLD and can be reversed by treatment. About 10–20% of simple fatty liver develops into NASH [37]. At present, NASH is an important link in the development of NAFLD for end-stage liver disease, such as cirrhosis, hepatocellular carcinoma, and liver failure [38]. Therefore, acetylation modification of proteins that are involved in metabolic pathways may be responsible for the increasing severity of fatty liver disease.

### Acetylation of proteins locates in the mitochondria contributes to the development of fatty liver disease in dairy cows

Since lysine acetylation is an important post-translational modification of proteins, it has been extensively studied for 50 years for its regulatory mechanisms in animals [8]. Proteins can gain or remove its enzymatic activity or specificity to certain substrates via acetylation or deacetylation, thereby regulating the energy and substance metabolism. Currently, However, little is known about the protein acetylome of fatty liver disease caused by negative energy balance in dairy cows, while there are relatively more studies in human and mouse liver tissues [39]. In this study, acetylome profiling of fatty livers revealed that 46.6% of the differentially expressed acetylated sites were localized in mitochondria, while 84.3% of the higher acetylated sites were localized to the mitochondria (Fig. 2), suggesting that the acetylation modification of hepatic proteins located in mitochondria plays a crucial role in both lipid and amino acid metabolism disorder in dairy cows, thereby contributes to the pathogenesis of fatty liver disease. In recent years, mitochondrial protein acetylation has received more attention. A study disclosed that 63% of mouse mitochondrial proteins contained lysine acetylation sites [40], devoting to abnormal mitochondrial oxidation of fatty acids, which is responsible for the degeneration of fatty liver disease [16]. Another study in mice showed that a high-fat diet could cause the hyperacetylation of liver mitochondrial oxygen metabolism-related proteins, leading to decreased activity of respiratory chain complexes [41]. It is presumed that mitochondrial protein acetylation would necessarily be considered as a potentially critical component of the mitochondrial metabolic regulatory network.

Actually, mitochondria are crucial for cellular energy metabolism processes, including the production of more than 90% of cellular ATP (respiratory chain), tricarboxylic acid cycle, β-oxidation, apoptosis, cell-cycle progression, proliferation and aging, and their dysfunction has been implicated in a wide range of metabolic diseases [36, 42–44]. Recently, a significant increase in protein lysine acetylation was found in pasture-fed Holstein-Friesian dairy cows during their early lactation period, accounting for impaired hepatic mitochrondrial function [45]. Low expression of sirtuin 1 (SIRT1), a NAD-dependent histone deacetylase, was indicated to promote hepatic fatty acid synthesis and inhibit fatty acid β-oxidation in dairy cows with mild fatty liver disease [46]. Histone acetyltransferses (HATs) and histone deacetylase (HDACs), as a senor of environmental nutrient and/or energy, balance the acetylation level of target proteins. Mammalian sirtuins (SIRTs) is an important type of NAD-dependent histone deacetylase, such as SIRT1 and SIRT3, were involved in oxidative stress and lipid metabolism regulation [47], and had been proposed as a reliable biomarker and/or therapeutic target for fatty liver disease [45–49]. It suggests that acetylation of mitochondrial protein was an emerging and fundamental mechanism regulating the development of fatty liver disease in dairy cows, through modifying the activities of mitochondrial proteins and overall mitochondrial function [47–49]. The mechanism how these acetylated proteins to be modified and their molecular function are involved in the pathogenesis of fatty liver disease will be necessarily elucidated in the future.

### Identification of candidate mitochondrial proteins as important regulation targets during the pathogenesis of fatty liver

From the acetylated proteins, it was observed that some mitochondrial proteins are involved not only in the fatty liver-related disorder protein network but also in several lipid metabolism-related pathways (Table 3). Therefore, it was speculated that acetylation of these proteins plays a key role in the development of fatty liver disease of dairy cows. Especially, most of the mitochondrial proteins were achieved a higher acetylation level (Tables 2 and 3). For example, carbamoyl phosphate synthase (CPS1) is the catalytic enzyme of the first step in the urea cycle, which is very important for the removal of excess urea from cells. Previous proteomic studies on non-alcoholic fatty liver showed that serum concentration of CPS1 decreases gradually in the order of control steatosis and NASH patient subjects and CPS1 has been confirmed to be serum candidate markers of NAFLD [50]. Consistent with results in the present study, previous studies also have observed a significant upregulation of HADHA acetylation levels, modified by sirtuin 3 (SIRT3) [47], in the liver tissues of mice that were fed with a high-fat diet for 1 week [51]. Activation of PPARα promotes lipolysis, and HADHA on the livers of hepatocyte humanized mice treated with PPARα agonists is significantly upregulated at the level of mRNA [52]. The level of HADHA in human renal cell carcinoma, breast cancer, and hepatocellular carcinoma is significantly downregulated [53–55]. Another new important candidate protein ACAT1 was identified, because of its ample interactions with other functional proteins and also plenty acetylation sites (Fig. 6, Table 2). Previous studies have confirmed that ACAT1 plays a catalytic role in ketone decomposition and formation, fatty acid oxidation, and isoleucine degradation [56]. ACAT1 is also associated with anti-cancer resistance, cancer cell proliferation, and cancer growth [57]. ACAT1 is the key enzyme catalyzing the synthesis of cholesterol ester at the last step. Cholesterol ester is one of the core components of lipid droplets [58–60]. Similarly, 3-hydroxy-3-methylglutaryl-CoA synthase 2 (HMGCS2) was identified as a potential crucial biomarker for dairy cows suffering from ketosis. HMGCS2 is an important mitochondrial enzyme of ketogenesis, which is a metabolic process that provides lipid-derived energy for various organs during the carbohydrate deprivation. The significantly abnormal acetylation level of HMGCS2 in fatty livers (Table 3) indicated a ketone metabolism disorder in dairy cows with fatty liver disease. In addition, the expression of EHHADH is regulated by PPARα, which is involved in the tricarboxylic acid cycle and peroxisome fatty acid oxidation [61]. Proteome results showed that cows with a high propensity to mobilize fat had greater whole-body fat oxidation compared to healthy cows [62]. In addition to mitochondrial β oxidation, fatty acid degradation in peroxisomes and microsomes increases proportionally. After the perinatal period, the fatty acid β oxidative capacity of peroxisomes decreases [63]. Thus, EHHADH, a fairly new candidate protein, was identified to be potentially associated with the pathogenesis of fatty liver disease, for its abundant interaction networks with other proteins (Table 3).

## Conclusions

In conclusion, this study provides a comprehensive acetylome profile of liver in dairy cattle and reveals important mitochondria-associated pathways that are involved in the regulation of the pathogenesis of fatty liver disease, including the TCA cycle, propionate metabolism, glycolysis/gluconeogenesis, pyruvate metabolism, oxidative phosphorylation, fatty acid degradation, valine, leucine, and isoleucine degradation, drug metabolism - cytochrome P450, and the PPAR signaling pathway. Furthermore, this study identified potential important proteins, such as HADHA, ACAT1, and EHHADH, which may be important regulatory factors being acetylation level modified in the development of fatty liver disease in dairy cows, potentially being therapeutic targets for NAFLD in human beings. However, further investigation is needed to focus on its molecular mechanism of regulating the hepatic metabolism. In the future, proteomic analyses would be necessary to combine with acetylomic analyses, so as to further uncover the important regulatory function of acetylation modification on liver function.

## Methods

### Ethics statement

All animal experiments were carried out according to the Regulations for the Administration of Affairs Concerning Experimental Animals published by the Ministry of Science and Technology, China (2004), and were approved by the Shandong Agricultural University Animal Care and Use Committee (approval number, SDAUA-2017-044).

### Sample collection

Dairy cows at 7 ± 2 days (*n* = 24) post parturition, with similar body weights, body conformation condition scores, and less than three parity were selected for liver biopsy [5, 60]. The cows were housed in a free stall barn with constant access to water and feed at a farm affilated to Holstein Cattle Association in Shandong Province. Before the liver biopsy procedure, the dairy cows were held in a cage and shaved on their side at the intersection of the 10th–11th rib and the middle humerus to the hip tubercle with an area of 5 × 5 cm [45, 64] . After sanitization, subcutaneous injection of 500 mg of 0.5% procaine was provided for local anesthesia. Next, the skin was cut with an incision of 0.5–1 cm in length, followed by the liver biopsy process operated by a veterinary surgeon [34, 36]. The liver tissues were biopsied using the Bard Magnum biopsy system (Bard Peripheral Vascular, Inc., Tempe, AZ, US) followed by surgical suturing of the skin. The animal was administered ketoprofen and penicillin G procaine by intravenous injection immediately after biopsy. The same injection protocol was performed for the next 3 to 5 days, and the animal was monitored for up to 2 weeks depending on its condition until complete recovery of health. Liver tissue samples were immediately snap-frozen in liquid nitrogen for later analysis or fixed in 5% polyformaldehyde for oil red O staining.

The biopsied dairy cattle had their fatty liver condition diagnosed according to the average percentage of hepatic cells containing lipid droplets in the tissue, and other background information of these cows including production and serum biochemical indexes [1, 60] was shown in Table S2. As a result, six samples were diagnosed as normal liver (6.26 ± 5.23%) and eight as severe fatty liver (86.75 ± 4.83%). These normal liver samples (Norm) and/or fatty liver samples (FL) were randomly re-grouped in an equal proportion for acetylome analysis, named as Norm1, Norm2, Norm3, and FL1, FL2, FL3, respectively.

### Protein extraction and trypsin digestion

The sample was retrieved from − 80 °C conditions, an appropriate amount of tissue sample was weighed into a liquid nitrogen pre-cooled mortar, and the liquid nitrogen was sufficiently ground to a powder. Each group of samples was separately added to a powder of four volumes of lysis buffer (8 M urea, 1% protease inhibitor, 3 μM TSA, 50 mM NAM, and 2 mM EDTA) and sonicated. After centrifugation at 12,000 g for 10 min at 4 °C, the cell debris was removed, the supernatant was transferred to a new centrifuge tube, and the protein concentration was determined using a BCA kit.

For digestion, the protein solution was reduced with 5 mM dithiothreitol for 30 min at 56 °C and alkylated with 11 mM iodoacetamide for 15 min at room temperature in darkness. The protein sample was then diluted by adding 100 mM TEAB to urea with a concentration of less than 2 M. Finally, trypsin was added at a ratio of 1:50 trypsin-to-protein mass for the first digestion overnight and 1:100 trypsin-to-protein mass ratio for a second 4 h digestion.

### TMT labeling and HPLC fractionation

After trypsin digestion, the peptide was desalted by Strata X C18 SPE column (Phenomenex) and vacuum dried. The peptide was reconstituted in 0.5 M TEAB and processed according to the manufacturer’s protocol for the TMT kit. The tryptic peptides were fractionated by high pH reverse-phase HPLC using a Thermo Betasil C18 column (5 μm particles, 10 mm ID, 250 mm length).

### Affinity enrichment of lysine-acetylated peptides

The peptide segment was dissolved in the IP buffer solution (100 mM NaCl, 1 mM EDTA, 50 mM Tris-HCl, 0.5% NP-40, pH 8.0). The supernatant was transferred to the acetylated resin (PTM104, Hangzhou Jingjie Biotechnology Co., Ltd., PTM Bio), which was washed in advance, and incubated overnight at 4 °C on a shaker. After incubation, the resin was washed four times with IP buffer solution and then twice with deionized water. Finally, the resin-bound peptide segments were eluted with 0.1% trifluoroacetic acid eluent three times. The eluent was collected and vacuum freeze-dried. After drying, desalination was carried out according to C18 ZipTips instructions. After vacuum freeze-drying, the samples were analyzed by liquid chromatography-mass spectrometry (LC-MS).

### LC-MS/MS analysis

The tryptic peptides were dissolved in 0.1% formic acid (solvent A) and directly loaded onto a homemade reversed-phase analytical column (15 cm length, 75 μm, i.d.) at a constant flow rate of 400 nL/min on an EASY-nLC 1000 UPLC system. The gradient comprised solvent B with a series of treatments: 6 to 23% (0.1% formic acid in 98% acetonitrile) for 26 min, 23 to 35% for 8 min, and up to 80% for 3 min, followed by holding at 80% for the last 3 min.

The peptides were subjected to an NSI source followed by tandem mass spectrometry (MS/MS) in Q ExactiveTM Plus (Thermo) coupled online to the UPLC. The electrospray voltage was 2.0 kV. The m/z scan range was 350 to 1800 for a full scan, and intact peptides were detected in the Orbitrap at a resolution of 70,000. Peptides were then selected for MS/MS using the NCE setting as 28, and the fragments were detected in the Orbitrap at a resolution of 17,500. A data-dependent procedure that alternated between one MS scan followed by 20 MS/MS scans with 15.0 s dynamic exclusion was followed. Automatic gain control (AGC) was set at 5E4. The fixed first mass was set as 100 m/z.

### Database search

The resulting MS/MS data were processed using the Maxquant search engine (v.1.5.2.8). Tandem mass spectra were searched against the acetylation database concatenated with reverse decoy database. Trypsin/P was specified as a cleavage enzyme allowing up to four missing cleavages. The mass tolerance for precursor ions was set as 20 ppm in First search and 5 ppm in Main search, and the mass tolerance for fragment ions was set as 0.02 Da. Carbamidomethyl on Cys was specified as fixed modification, and acetylation modification and oxidation on Met were specified as variable modifications. FDR was adjusted to < 1%, and the minimum score for modified peptides was set at > 40.

Secondary mass spectrometry data were retrieved using Maxquant (v1.5.2.8). Retrieval parameter settings: UniProt *Bos Taurus* (24,215 sequences) was used as the database, anti-library was added to calculate the false positive rate (FDR) caused by random matching, and common contamination database was added to the database to eliminate the influence of contaminated proteins in identification results; restriction mode was set to Trypsin/P; number of missing sites was set to be Trypsin/P. 2. The minimum length of the peptide segment was set to seven amino acid residues; the maximum modification number of the peptide segment was set to five; the mass error tolerance of the primary parent ions of the First search and Main search was set to 20 and 5 ppm, respectively, and the mass error tolerance of the secondary fragment ions was 0.02 Da. Alkylation of cysteine was set as fixed modification, which could be changed into oxidation of methionine and acetylation of the N-terminal of protein. The quantitative method was set to TMT-6 plex, and the FDR of protein identification and PSM identification was set to 1%.

### Bioinformatics analysis

The functions and characteristics of these proteins are explained in detail from the aspects of Gene Ontology (GO), and the annotation proteome was derived from the UniProt-GOA database (http://www.ebi.ac.uk/GOA/). First, the identified protein ID was converted to UniProt ID and then mapped to GO IDs by protein ID. Then, proteins were classified by Gene Ontology annotation based on three categories: biological process, cellular component, and molecular function. Identified protein domain functional descriptions were annotated by InterProScan (a sequence analysis application) based on the protein sequence alignment method, and the InterPro domain database was used. The KEGG pathway database was used to annotate the protein pathway. First, the KEGG online service tool KAAS was used to annotate the submitted protein, and then match the annotated protein into the corresponding pathway in the database through the KEGG mapper. Wolfpsort, software for predicting subcellular localization, was used to annotate the subcellular localization of the submitted proteins. Soft motif-x was used to analyze the model of sequences constituted with amino acids in specific positions of modify-21-mers (ten amino acids upstream and downstream of the site, but phosphorylation with modify-13-mers have six amino acids upstream and downstream of the site) in all protein sequences. All the database protein sequences were used as background database parameters, and the other parameters were maintained at the default settings.

All sequences of differentially expressed proteins were searched against the STRING database (version 10.5) for protein-protein interactions. Only interactions between the proteins belonging to the searched dataset were selected, thereby excluding external candidates. STRING defines a metric called “confidence score”. To define the interaction confidence, all interactions that had a confidence score ≥ 0.7 (high confidence) were gathered. A graph of theoretical clustering algorithm, molecular complex detection (MCODE), was utilized to analyze densely connected regions.

For further hierarchical clustering based on differentially modified protein functional classification (such as GO, Domain, Pathway), all the categories obtained after enrichment were collated, along with their *P* values, and then filtered for those categories that were at least enriched in one of the clusters with *P*-value < 0.05. This filtered P-value matrix was transformed by the function x = −log10 (*P*-value). Finally, these x values were z-transformed for each functional category. These z scores were then clustered by one-way hierarchical clustering (Euclidean distance, average linkage clustering) in Genesis. Differentially expressed proteins were divided into four parts according to their differentially expressed multiples: Q1 (0 < Ratio ≤ 1/1.5), Q2 (1/1.5 < Ratio ≤ 1/1.2), Q3 (1.2 < Ratio ≤ 1.5), and Q4 (Ratio > 1.5). Cluster membership was visualized with a heat map using the “heatmap.2” function from the “gplots” R package.

## Supplementary information

**Additional file 1: Fig. S1.** Data reliability testing. **Fig. S2.** Gene Ontology (GO) functional annotation of differentially acttylated proteins at lysines. **Fig. S3.** Identification of highly enriched protein-protein interaction clusters.

**Additional file 2: Table S1.** List of differentially expressed acetylated proteins identified and quantified by the TMT analysis in the study.

**Additional file 3: Table S2.** Production information of normal (Norm) and fatty liver dairy cows that were liver biopsied and their serum biochemical parameters ^1^.

## Data Availability

The data used to support the findings of this study are available from the corresponding author upon request.

## Abbreviations

GO: Gene Ontology
KEGG: Kyoto Encyclopedia of Genes and Genomes
Kac: Lysine acetylated
DAPs: Differentially acetylated proteins
NEFA: Non-esterified fatty acids
HADHA: HADHA protein
ACAT1: Acetyl-CoA acetyltransferase
EHHADH: Enoyl-CoA hydratase and 3-hydroxyacyl CoA dehydrogenase
ANXA6: Annexin A6
CPS1: Carbamoyl-phosphate synthase 1
GOT2: Aspartate aminotransferase
HMGCS2: Hydroxymethylglutaryl-CoA synthase
HADHB: Trifunctional enzyme subunit beta
ACAA2: 3-ketoacyl-CoA thiolase
ACADM: Medium-chain specific acyl-CoA dehydrogenase
ACADVL: Very long-chain specific acyl-CoA dehydrogenase
PCK2: Phosphoenolpyruvate carboxykinase 2
IDH2: Isocitrate dehydrogenase (NADP)
MDH2: Malate dehydrogenase
SUCLG1: Succinate-CoA ligase (ADP/GDP-forming) subunit alpha
FABP1: Fatty acid-binding protein
SCP2: Non-specific lipid-transfer protein
TAG: Triglycerides
VLDL: Very-low-density lipoprotein
