# Supplementary material for: Protein acetylation in mitochondria plays critical functions in the pathogenesis of fatty liver disease
Source: BMC Genomics. 2020 Jun 26;21:435. doi: 10.1186/s12864-020-06837-y (PMC7318365; doi:10.1186/s12864-020-06837-y)
Supplement: Supplementary file 1 — Additional file 1: Fig. S1. Data reliability testing. Fig. S2. Gene Ontology (GO) functional annotation of differentially acttylated proteins at lysines. Fig. S3. Identification of highly enriched protein-protein interaction clusters. [file 12864_2020_6837_MOESM1_ESM.docx]

**Protein Acetylation in Mitochondria Plays Critical Functions in the Pathogenesis of Fatty Liver Disease**

**Authors**

Zhang Le-tian^§^, Hu Cheng-zhang^§^, Zhang Xuan, Qin Zhang, Yan Zhen-gui, Wei Qing-qing, Wang Sheng-xuan, Xu Zhong-jin, Li Ran-ran, Liu Ting-jun, Su Zhong-qu, Wang Zhong-hua, Shi Ke-rong**^*^**

**Affiliations**

College of Animal Science and Technology, Shandong Agricultural University, Shandong Key Laboratory of Animal Bioengineering and Disease Prevention, Taian, Shandong, 271018, P. R. China.


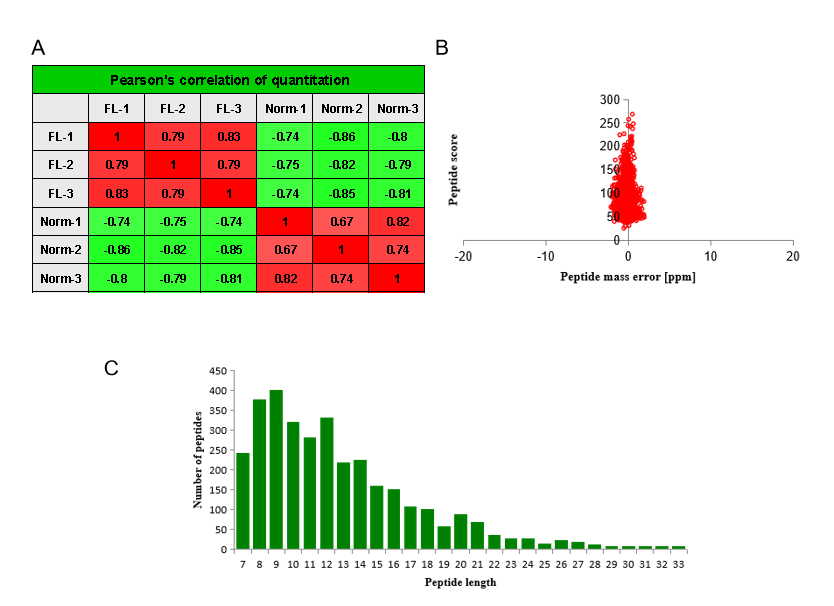


**Fig.S1 Data reliability testing.** (A) Pearson correlation coefficient between two pairs of samples. (B) Identification of the mass error distribution of the peptide segment. (C)Identification of the length distribution of the peptide segment.


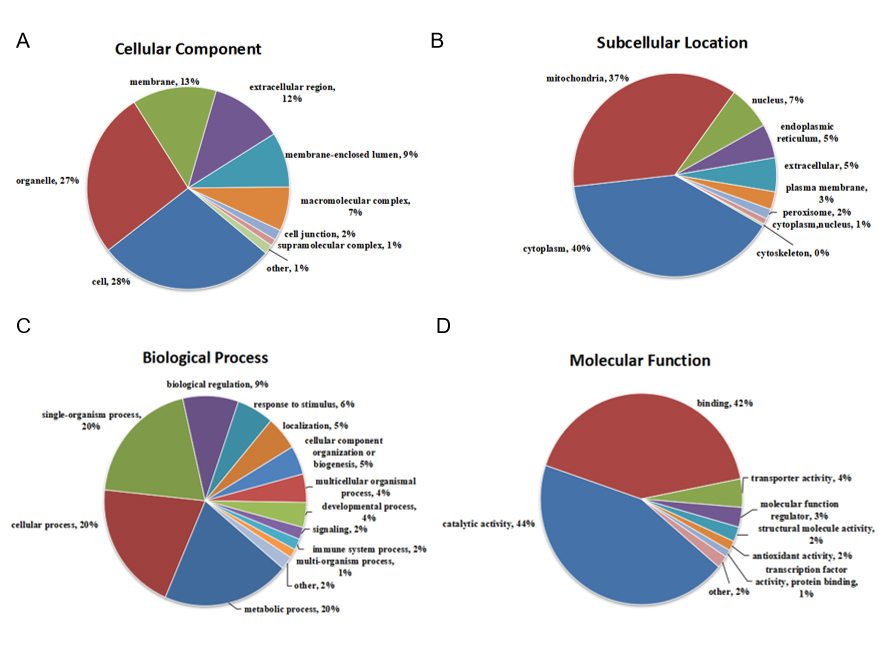


**Fig.S2** **Gene Ontology (GO) functional annotation of differentially acttylated proteins at lysines.** (A)Biological process. (B)Cellular component. (C)Molecular function. (D)Subcellular location.


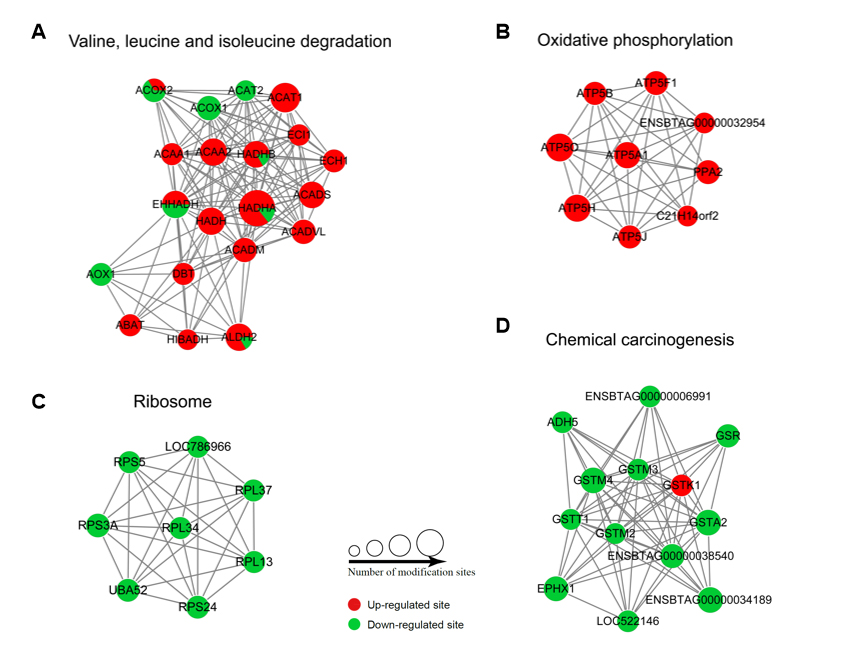


**Fig.S3 Identification of highly enriched protein-protein interaction clusters.** Four of clusters that showing differentially acetylated proteins were highly interacting each other, they are valine, leucine and isoleucine degradation (A), oxidative phosphorylation (B), ribosome (C) and chemical carcinogenesis (D). The size of the circle indicates the number of acetylation sites in the protein. The color of the circle indicates the modified directions of the acetylated sites in the protein, red presents higher-acetylated sites and greed presents lower-acetylated sites.
