# Supplementary material for: Protein acetylation in mitochondria plays critical functions in the pathogenesis of fatty liver disease
Source: BMC Genomics. 2020 Jun 26;21:435. doi: 10.1186/s12864-020-06837-y (PMC7318365; doi:10.1186/s12864-020-06837-y)
Supplement: Supplementary file 3 — Additional file 3: Table S2. Production information of normal (Norm) and fatty liver dairy cows that were liver biopsied and their serum biochemical parameters 1. [file 12864_2020_6837_MOESM3_ESM.docx]

**Table S2 Production information of normal (Norm) and fatty liver dairy cows that were liver biopsied and their serum biochemical parameters ^1^**

| **Items** | **Norm ^2^（n=6）** | **FL ^2^（n=8）** | ***P*-value** |
| --- | --- | --- | --- |
| Fat percentage in liver (%) **^3^** | 6.26±8.20 | 86.75±4.83 | 2.59E-11 |
| Parity No. | 1.17±0.41 | 1.38±0.74 | 0.55 |
| Dry matter intake (kg/d) | 20.6±2.3 | 19.4±3.0 | 0.22 |
| Day in milk (day) | 7±2 | 7±2 | 1.00 |
| Body weight (kg) | 650±53 | 668±45 | 0.34 |
| Milk yield (kg/d) | 26.2±1.9 | 25.3±3.2 | 0.67 |
| INS (pg/ml) | 171.37±26.46 | 268.34±183.83 | 0.45 |
| NEFA(mmol/L) | 1.60±0.88 | 2.78±1.74 | 0.16 |
| BHB (mmol/L) | 78.55±10.64 | 73.54±28.04 | 0.79 |
| GLU (mmol/L) | 3.05±0.64 | 1.93±0.72 | 0.011 |
| AST (IU/L) | 92.83±50.94 | 178.75±116.39 | 0.12 |
| TP (g/L) | 72.17±13.39 | 63.98±7.98 | 0.18 |
| ALB (g/L) | 28.88±3.76 | 29.48±3.44 | 0.76 |
| SUN (μmol/L) | 3.09±0.73 | 4.41±2.39 | 0.22 |
| UA (mol/L) | 36.83±7.63 | 31.63±10.65 | 0.33 |
| TG (μmol/L) | 0.13±0.03 | 0.10±0.03 | 0.19 |
| TCHO (μmol/L) | 2.01±0.64 | 1.63±0.29 | 0.16 |

^1^ INS, insulin; NEFA, non-esterified fatty acid; BHB, β-hydroxybutyric acid; GLU, glucose ; AST, aspartate aminotransferase; TP, total protein; ALB, serum albumin; SUN, serum urea nitrogen; UA, urea acid; TG, triglyceride; TCHO, total cholesterol.

^2^ Norm, normal cows; FL, fatty liver cows.

^3^ It shows the averaged percentage of cells containing lipid droplets in liver tissue, which indicated by Oil Red staining.
